# Wheel of misfortune: A unique case of MRSA pyomyositis of the adductor muscle group from blunt unicycle trauma

**DOI:** 10.1002/ccr3.8295

**Published:** 2023-12-17

**Authors:** Jordan I. Gaelen, Toluwalase Awoyemi, Emmanuel Okematti, Meera Ramanathan

**Affiliations:** ^1^ Department of Medicine Northwestern University Feinberg School of Medicine Chicago Illinois USA

**Keywords:** echocardiography, MRSA, pyomyositis, trauma

## Abstract

In patients with infectious symptoms and severe muscle pain, it is crucial to consider pyomyositis as a significant potential cause. A normal complete blood count should not exclude this possibility early in the course. Early advanced imaging modalities and blood cultures are crucial in narrowing the differential. Methicillin resistant *Staphylococcus aureus* is increasingly implicated.

## Graphical abstract

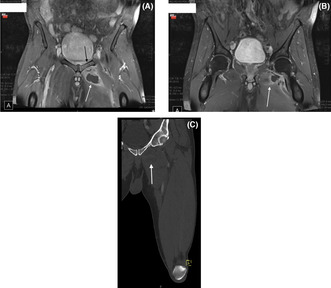

## 1 INTRODUCTION

Pyomyositis is a predominantly tropical pediatric disease typically caused by methicillin sensitive *Staphylococcus aureus*, though the incidence of methicillin resistant *Staphylococcus aureus* (MRSA) is on the rise.^1^, ^2^ It is often preceded by blunt trauma, but the inoculating source may not be found in 50–70% of cases.^3^ The quadriceps and glutei muscles are the most reported sites, with a few case reports detailing unconventional sites such as the obturator and the abductor groups of muscles.^1^, ^4^, ^5^, ^6^, ^7^, ^8^ To our knowledge, this is the first described case of MRSA pyomyositis in the adductor group of muscles. The leading theory for pathogenesis is the inoculation of MRSA into the blood stream following penetrating trauma, followed by direct involvement of muscle groups at the site of the trauma or indirect seeding of MRSA to susceptible muscle groups, such as traumatized muscles with intramuscular hematoma.

In this case report, we present a rare case of MRSA pyomyositis in the adductor muscle group complicated by septic arthritis and osteomyelitis in a young healthy patient with a history of blunt trauma. We highlight the need for a high index of suspicion for bacterial pyomyositis compared to other forms of myositis, the importance of sequential multimodal imaging in evaluating infectious musculoskeletal etiologies caused by MRSA, and the importance of infection source control.

## 2 CASE PRESENTATION

**FIGURE 1:**
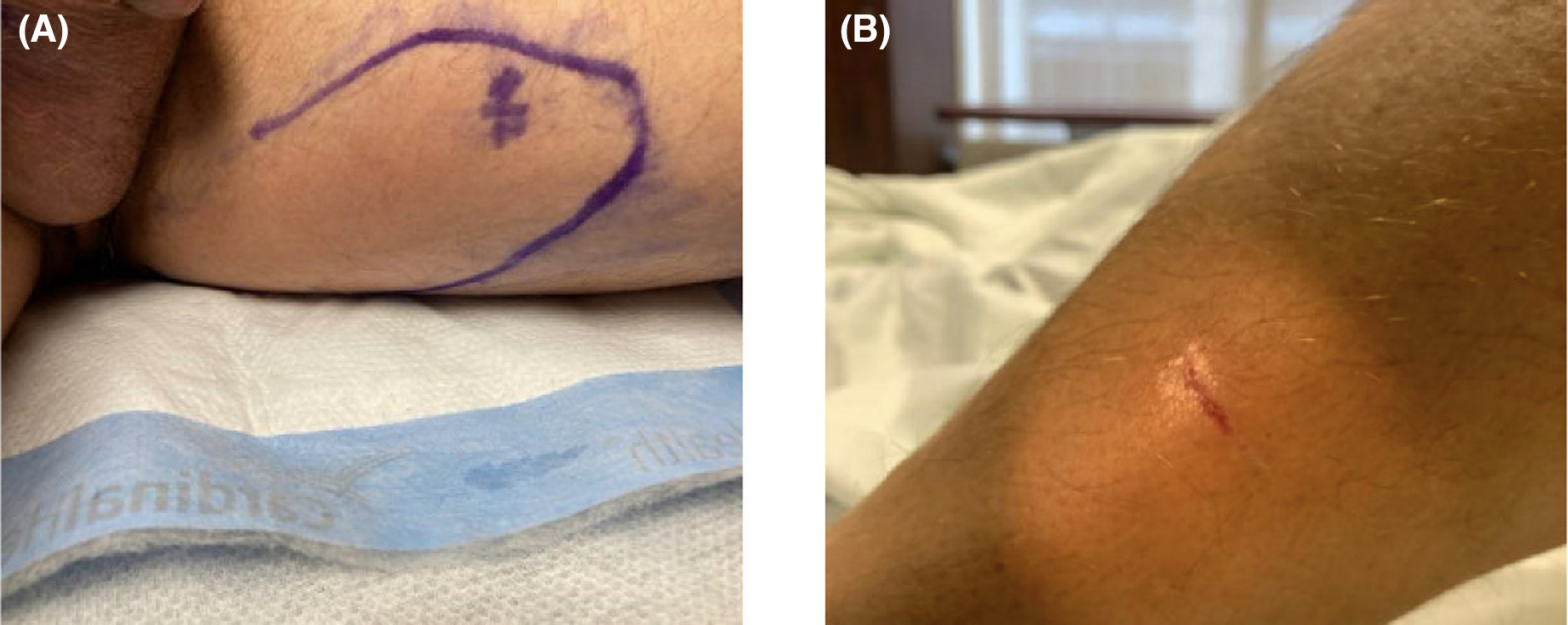
(A) Area of erythema and edema corresponding to area of inguinal pain. (B) Abrasion on lateral left lower leg which may be site of MRSA inoculation. The circle around the abrasion is from a light source.

A male patient, aged 53, with no prior medical history or history of diabetes presented with a 6‐day history of worsening left inguinal pain associated with weakness, rigors, subjective fever, and mobility impairment following blunt trauma to the groin (the trauma occurred due to the patient jumping from a motorized seatless unicycle). On assessment at the emergency department he was hemodynamically stable (blood pressure: 110/62, pulse: 98 beats per minute, respiratory rate: 18 cycles per minute) with a maximum temperature of 39.4°C. His left inguinal region revealed some mild faint erythema, swelling (Figure 1A) and reproducible pain worsened with movement. The patient also had an abrasion of about 1.5 cm in diameter on the lateral left lower leg (Figure 1B). On questioning, this abrasion was about a month old and was the result of an injury sustained while gardening.

**FIGURE 2:**
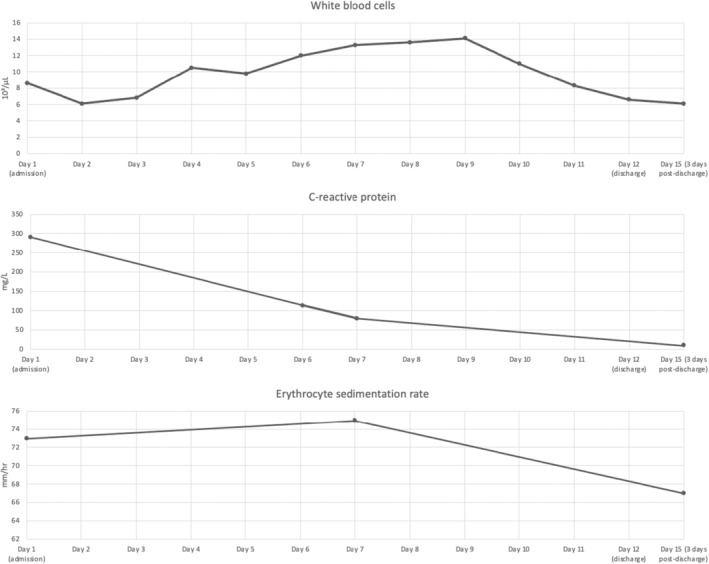
Infectious and inflammatory markers over course of admission. White blood cells (ref. 3.5–10.5 × 10^3^/μL). C‐reactive protein (ref. 0.0–10.0 mg/L). Erythrocyte sedimentation rate (ref. 3–10 mm/h).

**FIGURE 3:**
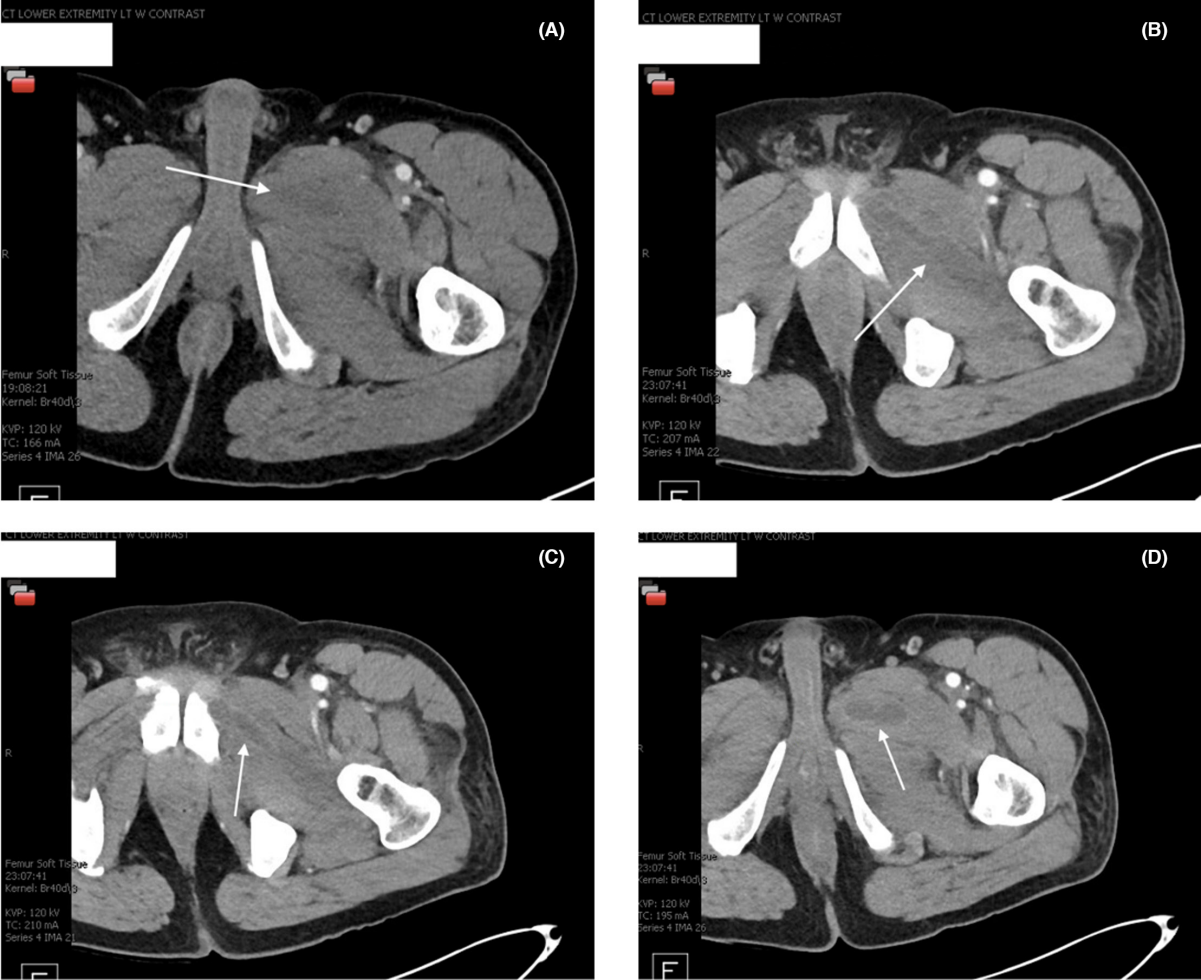
(A, B) Computed tomography (CT) with contrast of lower extremity showing evidence of myositis. (C, D) Repeat CT with contrast of lower extremity showing abscesses.

Initial laboratory investigation revealed elevated creatinine kinase (CK) (970 IU/L), procalcitonin (2.038 ng/mL), erythrocyte sedimentation rate (ESR) (75 mm/h), C‐reactive protein (CRP) (291.6 mg/L), abnormal urinalysis (3+ blood without red blood cells), positive nasal swab for MRSA colonization, and positive blood cultures (MRSA grew within the first 24 h). Complete blood count (CBC) was notable for normal leukocyte counts (8.6 × 10^3^/μL) (See Figure 2 for inflammatory markers over the course of treatment). The complete metabolic panel was notable for mildly elevated creatinine of 1.57 mg/dL. Viral (Coxsackie A, echovirus, human immunodeficiency virus (HIV), and parvovirus B19) and autoimmune antibody panels were all negative. Computed tomography (CT) of the lower extremity revealed diffuse asymmetric edema and hypoenhancement of the left obturator externus and adductor muscles, suspicious for myositis (Figure 3A,B).

He was started on empiric intravenous (IV) vancomycin and piperacillin‐tazobactam which was subsequently de‐escalated to vancomycin based on microbiological susceptibility results. A transthoracic echocardiogram (TTE) was normal but transesophageal echocardiogram (TEE) showed a small, filamentous, mobile structure on the atrial aspect of the anterior leaflet of the mitral valve which could have represented degenerative leaflet stranding, though infective endocarditis could not be excluded (Video S1). Trivial mitral regurgitation was seen. There were no concerning features such as recurrent systemic embolization, severe heart failure, severe valve dysfunction, invasion beyond the valve leaflets, antibiotic resistant infective endocarditis, or large mobile vegetations that would have warranted surgical management. Despite prompt commencement of IV vancomycin, the patient's clinical condition failed to improve, with persistent fevers up to 38.7°C, progressive inguinal pain, and worsening mobility impairment, necessitating a repeat CT scan of the lower limb to re‐evaluate the myositis.

Repeat CT scan showed two additional abscesses not previously captured by the preceding CT scan (Figure 3C,D). The abscesses were percutaneously drained but the aspiration yielded a minimal amount of fluid, despite the presence of evidence on the CT scan. The cultured aspirate yielded MRSA. Despite abscess drainage, the patient's symptoms persisted with high‐grade fevers, worsening pain, and uptrending white blood cell (WBC) counts.

**FIGURE 4:**
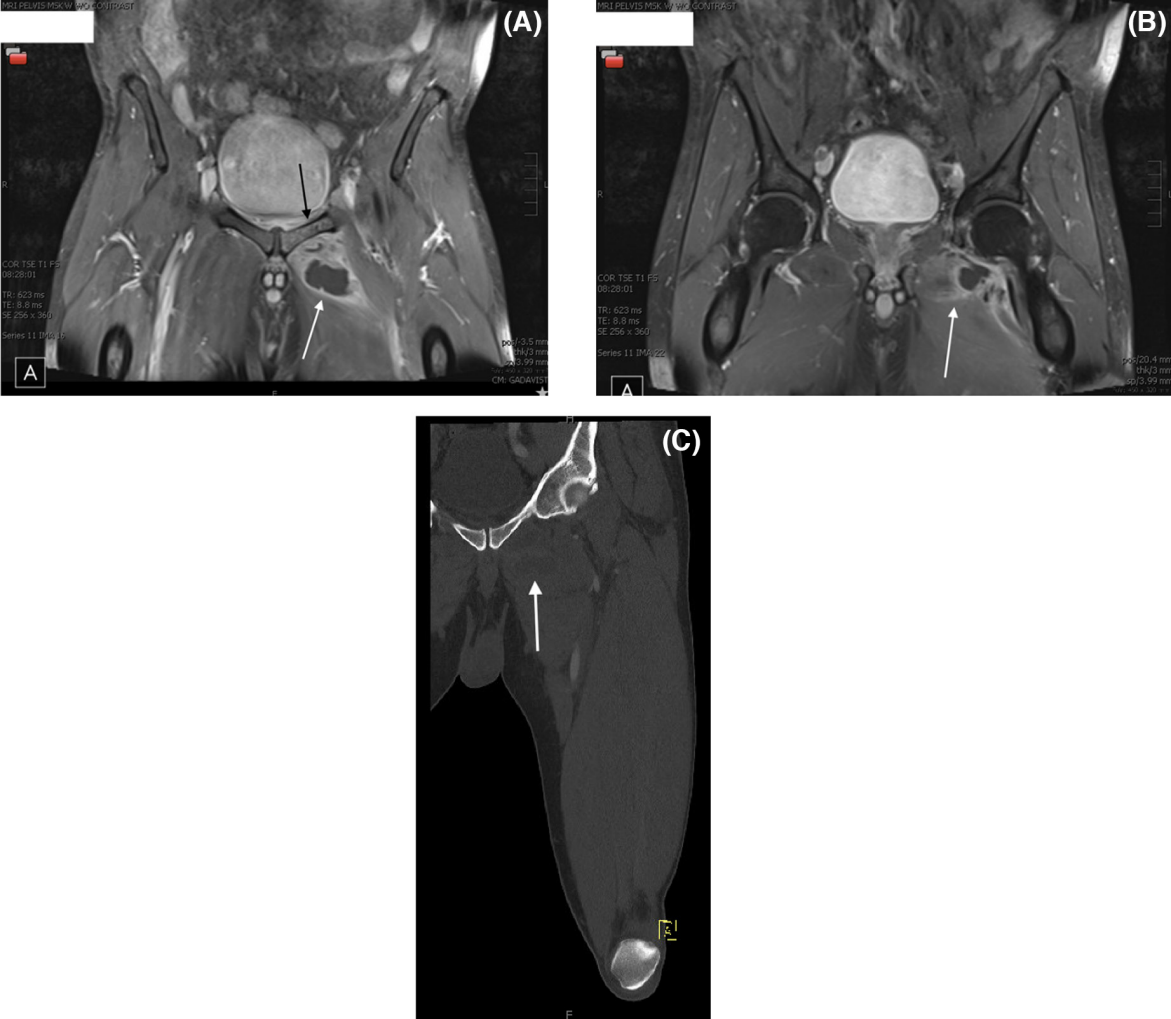
(A–C) Magnetic resonance imaging (MRI) with and without contrast of the pelvis showing recurrent abscesses (white arrow), with new findings concerning for pelvic osteomyelitis (black arrow).

He was re‐imaged with a magnetic resonance imaging (MRI) scan of the pelvis which showed recurrent and larger abscess, measuring 8.5 cm, in the left adductor longus and brevis, acute pubic bones osteomyelitis, and pubic symphysis septic arthritis (Figure 4A–C). The abscess was drained and an external drain placed. Cultured aspirate grew MRSA. Subsequently, the patient's symptoms improved, and his CRP began to downtrend. Following remarkable clinical improvement, return to baseline mobility, and two consecutive negative blood cultures drawn on Day 4 of admission, the patient was discharged to complete 6 weeks of IV vancomycin through a peripherally inserted central catheter (PICC) line. His drain was removed 4 days after discharge. Upon discharge, he exhibited residual deficits, specifically weakness in hip extension and adduction, albeit significantly improved compared to admission. Remarkably, these deficits completely resolved within 3 months with the aid of weekly physical therapy. As of the time of writing this case report (5 months post‐discharge), there are no lingering residual deficits. A follow‐up TEE conducted 2 months after discharge revealed findings that were non‐concerning for infective endocarditis.

## 3 DISCUSSION

MRSA pyomyositis is an unusual infection in adults, but it is becoming increasingly prevalent likely due to increasing prevalence of comorbid conditions such as diabetes mellitus, obesity, HIV infection or changing bacterial characteristics.^1^, ^2^, ^9^ Its pathogenesis remains unclear, with one theory being the presence of muscle trauma in the setting of transient bacteremia allowing for an infection to seed in the injured muscle.^10^ Trauma is consistently cited as the most important risk factor in the eventual development of pyomyositis, with blunt trauma being the primary mechanism.^1^, ^11^ Cases of pyomyositis as a result of sharp trauma, in particular injection drug use, have been described.^12^ Pyomyositis progresses in three stages, with a preliminary invasive stage that lasts 10–21 days characterized by localized edema, an insidious, dull pain, and low‐grade fevers. The second stage is characterized by purulence which lasts 1–12 days. The third stage is characterized by systemic symptoms, metastatic abscesses, and multi‐organ dysfunction.^13^ Given the transient nature of this condition's bacteremia, blood culture sensitivity is typically poor, with positive blood culture results in 5–38% of cases.^2^, ^7^, ^11^, ^14^ In patients that present with muscle weakness and pain due to the vague nature of the symptoms, it is necessary to have an extensive list of differentials such as infectious (bacterial or viral) or autoimmune myositis, septic arthritis, rhabdomyolysis, muscle strain, and osteomyelitis.

In our patient, the presence of high‐grade fevers, differential warmth, elevated procalcitonin, and rapid deterioration in mobility despite a normal leukocyte count heightened our suspicion for a highly virulent bacterial infection. We particularly considered viral and autoimmune myositis, which can often present similarly.^15^ However, viral myositis is often preceded by a viral prodrome which was absent in our patient.^15^ While it is not always possible to identify a specific virus in every patient who exhibits symptoms of a viral illness, it is worth noting that viral myositis can occasionally lead to the development of rhabdomyolysis.^16^, ^17^ In the United States, myositis is frequently linked to Influenza A and B viruses, as well as enteroviruses.^18^ However, additional viruses, including Coxsackievirus and Epstein–Barr virus (EBV), have also been identified as potential causes.^16^, ^17^ The absence of viral prodrome and negative viral studies made viral myositis less likely. Similarly, despite an elevated CRP, ESR, and CK, we felt an autoimmune myopathy was unlikely due to his asymmetric presentation, high grade fevers, and absence of joint manifestations and other multisystemic symptoms.^19^ Autoimmune myopathies encompass a diverse group of disorders, namely dermatomyositis, immune‐mediated necrotizing myopathy, antisynthetase syndrome, and polymyositis. Individuals diagnosed with autoimmune myopathy commonly exhibit bilateral weakness in the muscles closest to the center of the body, which worsens gradually over a period of weeks or months.^20^ Consequently, we obtained blood cultures among our initial lab work while the patient was still in the emergency department, allowing us to quickly identify and address an underlying bacteremia.

The detection of MRSA in the blood culture, along with the absence of viral and autoimmune antibody titers, heightened our suspicion for pyomyositis and led us to further investigate for an inoculation site. We therefore recommend early blood cultures in patients with a history of trauma; however, minor it may appear, presenting with a septic picture. The occurrence of muscular trauma or injury has been suggested as a potential precursor to the onset of pyomyositis. However, several investigations have indicated that a history of trauma or muscle damage is documented in only 30–50% of pyomyositis cases.^3^ The occurrence of myositis as a result of bacteremia is improbable in the absence of associated muscle injury. In one study examining staphylococcal bacteremia, it was observed that less than 0.5% of the participants developed myositis.^21^ In our patient's case, the pyomyositis was triggered by a minor blunt muscular trauma from jumping from his motorized unicycle creating a susceptible focus for infection. It was also surprising that within a few days of infection, he had evidence of infective endocarditis which led us to suspect that the laceration on his left leg, which happened a few weeks prior, potentially led to MRSA bacteremia with subsequent seeding.

Our case further supports the prior research conducted by Olson^2^ (2011), De Silva^7^ (2021), and Ovadia^23^ (2007), which indicates that the WBC count may seem to be within the normal range during the initial stages of pyomyositis. Therefore, it is important to note that a normal WBC count should not be used as a sole criterion for ruling out an infectious cause. In this particular instance, the WBCs effectively monitored the patient's advancement, as seen by a decrease in count from 11.0 to 8.3 on the subsequent day following the second drainage procedure. ESR remained stable throughout the hospital stay and after discharge, and therefore may not be a reliable biomarker for monitoring disease progression or treatment response. However, an elevated ESR may be useful in establishing a differential, as a normal ESR may help to exclude pyomyositis. Likewise, an elevated ESR may help to exclude viral myositis, where it is typically normal or only slightly elevated.^22^ CRP has been previously described as the most sensitive marker of disease progression and treatment response.^7^, ^23^ While we did not track CRP throughout the hospital stay, it is reassuring that his CRP down trended dramatically after treatment began and after discharge.

This case reinforces the importance of pursuing an extensive investigation in patients with an identified MRSA bacteremia. MRSA bacteremia should necessitate a search for sites of distant infection. In our patient we performed an initial TTE to investigate for infectious endocarditis which failed to identify any valvular lesions. However, given the propensity of MRSA to cause valvular vegetations and the superior sensitivity of a TEE, we decided to pursue a TEE, which identified potential infective endocarditis.^24^ Opting for a TTE as the initial diagnostic approach is justified by its lower cost, absence of sedation requirement, reduced risk of aspiration, and technical simplicity. If the TTE yields negative results, a subsequent TEE becomes imperative for conclusive exclusion of infective endocarditis. This is because TEE offers significantly higher sensitivity (90–100%) for native valve infectious endocarditis compared to the lower sensitivity range of 40–63% associated with TTE.^25^ TTE can serve as the sole investigation if it identifies valvular vegetations indicative of infective endocarditis. We also identified acute osteomyelitis and septic arthritis on MRI. In our patient, we initially opted for a CT scan due to uncertainty about the make‐up of a previous facial implant. However, we opted for an MRI once it was confirmed his implant was silicon and to evaluate for additional abscesses that may have not been captured on CT. Review of his initial CT imaging failed to demonstrate signs of septic arthritis or osteomyelitis, potentially due to the limitations of radiography to identify these sequelae early.^26^ Consequently, we agree with previous reports that identify MRI as the most sensitive imaging modality for pyomyositis.^1^, ^2^, ^6^, ^23^ We recommend MRI, if possible, as the diagnostic imaging of choice due to its temporal and spatial tissue characterization abilities. In cases where the patient's response to IV antibiotics is insufficient, repeat MRI imaging is crucial, given the infection's swift progression and potential for rapid dissemination to distant sites.

In our case it was difficult to achieve initial source control, requiring repeated imaging and an external drain placement. We believed the initial course of IV vancomycin was working given the patient noted increasing mobility and reduced pain. Repeat blood cultures 4 days after treatment initiation failed to grow MRSA. Daptomycin could have been considered as a secondary antibiotic had improvement not been noted.^24^, ^27^ A 6‐week course of antibiotics was decided upon given the patient's concomitant septic arthritis and contiguous osteomyelitis. Given this, we opted not to further assess the possible infectious endocarditis, given the treatment for a confirmed endocarditis would also have been 6 weeks of treatment, which was now considered redundant. In our patient, we achieved source control through repeated abscess aspiration and external drain placement. His drain was removed when the effluent was less than 10 mL/day for 3 days, based on recommendations by our interventional radiology colleagues.

We have presented an unusual case of pyomyositis in the adductor muscle group caused by methicillin‐resistant *Staphylococcus aureus* in a patient with no preceding penetrating trauma and discuss the importance of sequential multimodal imaging in evaluating infectious musculoskeletal etiologies caused by MRSA.

## AUTHOR CONTRIBUTIONS

**Jordan Gaelen:** Conceptualization; investigation; methodology; visualization; writing – original draft; writing – review and editing. **Toluwalase Awoyemi:** Conceptualization; investigation; methodology; validation; visualization; writing – original draft; writing – review and editing. **Emmanuel Okematti:** Conceptualization; investigation; methodology; validation; writing – review and editing. **Meera Ramanathan:** Conceptualization; investigation; methodology; supervision; validation; writing – review and editing.

## FUNDING INFORMATION

None.

## CONSENT

Written informed consent was obtained from the patient to publish this report in accordance with the journal's patient consent policy.

## Supporting information


Video S1.

## Data Availability

Data sharing is not applicable to this article as no new data were created or analyzed in this study.
